# Patients’ views on the implementation of artificial intelligence in radiology: development and validation of a standardized questionnaire

**DOI:** 10.1007/s00330-019-06486-0

**Published:** 2019-11-08

**Authors:** Yfke P. Ongena, Marieke Haan, Derya Yakar, Thomas C. Kwee

**Affiliations:** 1 Center of Language and Cognition, University of Groningen, Oude Kijk in ’t Jatstraat 26, NL 9700 AS Groningen, The Netherlands; 2 Department of Sociology, University of Groningen, Grote Rozenstraat 31, NL 9712 TG Groningen, The Netherlands; 3 Department of Radiology, Medical Imaging Center, University Medical Center Groningen, University of Groningen, Groningen Hanzeplein 1, NL 9713 GZ Groningen, The Netherlands

**Keywords:** Artificial intelligence, Surveys and questionnaires, Patients, Radiology

## Abstract

**Objectives:**

The patients’ view on the implementation of artificial intelligence (AI) in radiology is still mainly unexplored territory. The aim of this article is to develop and validate a standardized patient questionnaire on the implementation of AI in radiology.

**Methods:**

Six domains derived from a previous qualitative study were used to develop a questionnaire, and cognitive interviews were used as pretest method. One hundred fifty-five patients scheduled for CT, MRI, and/or conventional radiography filled out the questionnaire. To find underlying latent variables, we used exploratory factor analysis with principal axis factoring and oblique promax rotation. Internal consistency of the factors was measured with Cronbach’s alpha and composite reliability.

**Results:**

The exploratory factor analysis revealed five factors on AI in radiology: (1) distrust and accountability (overall, patients were moderately negative on this subject), (2) procedural knowledge (patients generally indicated the need for their active engagement), (3) personal interaction (overall, patients preferred personal interaction), (4) efficiency (overall, patients were ambiguous on this subject), and (5) being informed (overall, scores on these items were not outspoken within this factor). Internal consistency was good for three factors (1, 2, and 3), and acceptable for two (4 and 5).

**Conclusions:**

This study yielded a viable questionnaire to measure acceptance among patients of the implementation of AI in radiology. Additional data collection with confirmatory factor analysis may provide further refinement of the scale.

**Key Points:**

*• Although AI systems are increasingly developed, not much is known about patients’ views on AI in radiology.*

*• Since it is important that newly developed questionnaires are adequately tested and validated, we did so for a questionnaire measuring patients’ views on AI in radiology, revealing five factors.*

*• Successful implementation of AI in radiology requires assessment of social factors such as subjective norms towards the technology.*

## Introduction

Artificial intelligence (AI) is expected to revolutionize the practice of radiology by improving image acquisition, image evaluation, and speed of workflow [1, 2]. More and more sophisticated AI systems are being developed for use in clinical practice [1, 2].

Importantly, unilateral development of AI systems from the perspective of the radiologist ignores the needs and expectations of patients who are perhaps the most important stakeholders. AI systems may need to fulfill certain preconditions for this technology to be embraced by society [3]. Patient preferences determine the boundaries within which an AI system should function. At present, however, little is known on patients’ views on the use of AI in radiology [3].

Implementation of AI in radiology is an example of the much broader concept of consumer health information technology (CHIT). CHIT refers to the use of computers and mobile devices for decision-making and management of health information between healthcare consumers and providers [4]. In order to measure patients’ acceptance of CHIT, several questionnaires have been developed [5, 6], using Davis’ widely accepted technology acceptance model (TAM [7, 8]). However, since patients are not active users in the setting of AI in radiology, there is a need for a new method to measure technology acceptance when the patient is not actively using the technology, but is subjected to it.

To the best of our knowledge, there are no validated standardized questionnaires available for mapping patients’ views on the implementation of AI in radiology. The aim of this study was therefore to develop and, by means of expert evaluation, qualitative pretests, and factor analysis, validate a standardized patient questionnaire on the implementation of AI in radiology.

## Materials and methods

This prospective study was performed and approved by the local institutional review board of the University Medical Center Groningen (IRB number: 201800873), which is a tertiary care hospital that provides both primary and specialty care to approximately 2.2 million inhabitants in the Netherlands. All patients provided written informed consent.

### Questionnaire development

**Fig. 1:**
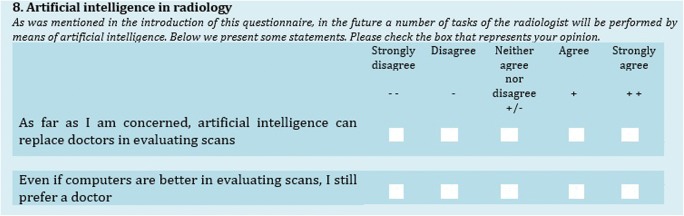
Layout of matrix with agree-disagree statements

To develop the questionnaire, we conducted semi-structured qualitative interviews with 20 participants in a previous study (see Haan et al [3]). Based on these interviews [3], six key domains of patients’ perspective on the implementation of AI in radiology were identified: proof of technology, procedural knowledge, competence, efficiency, personal interaction, and accountability. In the present study, we use these six domains as a framework for the questionnaire. Within each domain, a minimum of seven items, predominantly 5-point Likert-type agree-disagree scales, were developed. Using the rule of thumb that respondents answer about 4 to 6 items per minute [9], we limited the questionnaire to 48 attitudinal items (in 6 blocks of agree-disagree questions). We also used eight attitudinal items in an item-specific format. Since the response options in this format are content-related, the questions are assumed to require less cognitive processing and have shown to receive more conscientious responding [10]. In addition, an existing scale with adequate reliability (Cronbach’s alpha = 0.89) on orientation towards change [11] was used. We also included five demographic questions (birthdate, gender, education, digital device ownership and use), four yes-no questions on hypothetical situations, a check-all-that-apply question on trust, and one question asking participants to estimate the time range of implementation of AI in radiology practice. In accordance with general recommendations for paper-and-pencil questionnaires [12], we used a darker background to make answer boxes stand out (see Fig. 1).

### Questionnaire pretesting with cognitive interviews

A qualitative pretest of the first version questionnaire was done by means of cognitive interviews [13]. The main purpose of these interviews was first to ask participants to fill out the questionnaire, while thinking aloud. The interviewer probed after any cues of uncertainty of respondents. Seven graduate students in communication sciences, all with prior experience in interviewing, conducted a total of 21 interviews, based on a convenience sample with patients scheduled for a CT scan of the chest and abdomen on an outpatient basis. The 21 patients’ age ranged between 35 and 76 years (median, 63 years) and 11 of them were male. The interviews yielded several suggestions for improving the questionnaire. Firstly, from the cognitive interviews, it appeared that the difference between “agree” and “disagree” may be easily overlooked, and therefore, we added plus and minus signs (Fig. 1). Secondly, we adjusted terminology that was sometimes interpreted too general or too specific. In order to make it as clear as possible that questions are about AI replacing physicians specifically, we used the term “doctor” and “artificial intelligence” as often as possible in the questionnaire. Thirdly, we used shorter and clearer question wording for some statements by deleting superfluous wording such as “It is the question whether….”

### Procedure data collection

The patients for the quantitative data collection were recruited from December 19, 2018, until March 15, 2019. The patients that were scheduled for CT, MRI, and/or conventional radiography were approached by one of seven students in communication sciences (the same students that also conducted the cognitive interviews). All patients who were in the waiting room of our department for a radiological examination were approached. Based on the cognitive interviews, we estimated filling out the questionnaire would take about 15 min. We aimed for a sample with a subject to item ratio of at least 1:3 [14]. With 48 items in the original pool, this required a sample of at least 141. Sample size determination in exploratory factor analysis (EFA) is difficult, but with strong data, a smaller sample still enables accurate analysis. Following guidelines for “strong data” [14], we verified that none of the variable communalities (the proportion of each variable’s variance that can be explained by the factors) was lower than 0.40. We took 0.35 as a minimum factor loading and omitted items with cross-loadings higher than 0.50. Furthermore, we only included factors with more than 3 items.

### Data analysis

Data were analyzed by using IBM SPSS Statistics (Version 24). Exploratory factor analysis (EFA) was used to examine to which extent the items measured constructs related to AI in radiology, and to find underlying latent variables. In EFA, the relation between each item and the underlying factor is expressed in factor loadings, which can be interpreted similar to standardized regression coefficients. Principal axis factoring was used as the extraction method, since this method does not require multivariate normality. Oblique promax rotation was selected because correlations between factors were (somewhat) expected. The data were suitable for EFA as shown by Bartlett’s test of sphericity (< 0.001) and the Kaiser-Meyer-Olkin measure of sampling adequacy (0.719). The decision for the number of factors was made based on the Kaiser [15] criterion, a parallel analysis [16], and a scree test [17]. Items with low factor loadings were dropped (e.g., loading, < 0.35). Cronbach’s alpha was used to calculate the internal consistency of items within each factor. In general, Cronbach’s alpha of 0.7 is taken as indication of good internal consistency. In some cases, an alpha of 0.5 or 0.6 can be acceptable [18]. In order to overcome the disadvantages of Cronbach’s alpha (e.g., underestimation of reliability; see Peterson and Kim [18]), we also computed composite reliability in R [19]. This measure is interpreted similar to Cronbach’s alpha. In order to explore the meaningfulness of the factors that emerged from our factor analysis, we computed Pearson correlations with numerical demographic data (age and inclination to change) and performed analysis of variance for categorical demographics (gender and education).

## Results

### Sample

The respondents’ (*N* = 155) age ranged between 18 and 86 years (mean = 55.62, SD = 16.56); 55.6% of the respondents were male. 9.7% were educated at master or PhD level, 21.4% were at bachelor level, 24.0% were on mediate vocational level, 39.6% had high school level, and 5.2% had completed elementary-school-level education. There were several patients who indicated that they were not able to participate; in the far majority of cases, this was due to a lack of time (because of parking issues, work, or school-related activities, or because these patients had another scheduled appointment in the hospital).

### Results of EFA

The EFA generated five factors representing the following underlying latent variables: (1) “distrust and accountability of AI in radiology,” (2) “procedural knowledge of AI in radiology,” (3) “personal interaction with AI in radiology,” (4) “efficiency of AI in radiology,” and (5) “being informed of AI in radiology.”

Factors 1, 4, and 5 consist of a combination of items of the initial domains proof of technology, competence, and efficiency that were identified in our previous qualitative study [3]. Factors 2 (procedural knowledge) and 3 (personal interaction) correspond with the same domains as identified in the aforementioned qualitative study [3]. Originally, 17 items loaded on factor 1 “distrust and accountability.” Two items were dropped, to increase Cronbach’s alpha to 0.863. Originally, 6 items loaded on factor 4 “efficiency of AI in radiology,” which resulted in Cronbach’s alpha of 0.594. One item was dropped, to increase Cronbach’s alpha to 0.670. Five items, from the original domains accountability, procedural knowledge, and efficiency did not load on any factor and were therefore also dropped from the scale. For factor 5, Cronbach’s alpha remained just below 0.6. This factor includes items that do not directly assess the direction of attitude towards AI in radiology, and some items loaded negatively, which implies that items were not all positively correlated with the underlying variable. Moreover, in this case, we considered it better to not delete more items from this scale because the artificial effort to increase alpha above a certain level may harm reliability and validity [20]. In most cases, the composite reliability and Cronbach’s alpha were identical, but for factors 3 and 4, the composite reliability score was higher. Table 1 shows all the 39 items that remained for each of the 5 factors of the questionnaire. Table 2 shows the 8 items that were dropped from the questionnaire. We also verified correlations between factors, and concluded that none were strongly inter-correlated (Table 3). Factors 1 and 3 were moderately correlated, which indicates that patients value trust and accountability and personal interaction similarly.

**Table 1:** Descriptive figures of 39 attitudinal items for each of the 5 factors of the questionnaire

Attitudinal items	Mean^1^	Standard deviation	Factor loading
Factor 1 “distrust and accountability,” 15 items, Cronbach’s alpha 0.861, composite reliability 0.86			
Overall	3.28	0.584	–
1. A computer can never compete against the experience of a specialized doctor (radiologist)	3.43	0.874	0.677
2. Through human experience, a radiologist can detect more than the computer	3.37	0.896	0.668
3. Humans have a better overview than computers on what happens in my body	3.36	0.905	0.631
4. It worries me when computers analyze scans without interference of humans	3.68	0.971	0.605
5. I wonder how it is possible that a computer can give me the results of a scan	3.13	1.095	0.586
6. Artificial intelligence makes doctors lazy	2.56	0.995	0.579
7. I think radiology is not ready for implementing artificial intelligence in evaluating scans	3.14	0.681	0.568
8. I think replacement of doctors by artificial intelligence will happen in the far future	3.19	0.955	0.551
9. I would never blindly trust a computer	3.65	1.003	0.548
10. Artificial intelligence can only be implemented to check human judgment	3.53	0.906	0.517
11. I find it worrisome that a computer does not take feelings into account	3.97	1.078	0.475
12. It is unclear to me how computers will be used in evaluating scans	3.30	0.940	0.459
13. Even if computers are better in evaluating scans, I still prefer a doctor	3.32	1.041	0.410
14. When artificial intelligence is used, my personal data may fall into the wrong hands	3.32	0.981	0.397
15. Artificial intelligence may prevent errors^2^	2.88	0.930	0.365
Factor 2 “procedural knowledge,” 8 items, Cronbach’s alpha 0.927, composite reliability 0.93			
Overall	4.47	0.667	
1. I find it important to have a **good understanding**^3^ of the results of a scan	4.68	0.693	0.919
2. I find it important to be able to ask questions **personally** about the results of a scan	4.59	0.684	0.891
3. I find it important to **talk** with someone about the results of a scan	4.44	0.782	0.884
4. I find it important that a scan provides as **much information** about my body as possible	4.51	0.773	0.819
5. I find it important to get the results of a scan as **fast** as possible	4.49	0.805	0.802
6. I find it important to ask questions on the **reliability** of the results	4.42	0.843	0.725
7. I find it important to be **well informed** about how a scan is made	4.07	0.907	0.652
8. I find it important to **read** how radiologists work before I get a scan	3.63	1.005	0.467
Factor 3 “personal interaction,” 7 items, Cronbach’s alpha 0.777, composite reliability 0.82			
Overall	4.38	0.484	
1. When discussing the results of a scan, humans are indispensable	4.53	0.702	0.953
2. Getting the results involves personal contact	4.44	0.759	0.942
3. As a patient, I want to be treated as a person, not as a number	4.42	0.790	0.694
4. When a computer gives the result, I would miss the explanation	4.03	0.937	0.645
5. I find it important to ask questions when getting the result	4.59	0.575	0.449
6. Even when computers are used to evaluate scans, humans always remain responsible	4.35	0.780	0.391
7. Humans and artificial intelligence can complement each other	4.34	0.659	0.369
Factor 4 “efficiency,” 5 items, Cronbach’s alpha 0.670, composite reliability 0.69			
Overall	2.89	0.609	
1. As far as I am concerned, artificial intelligence can replace doctors in evaluating scans^2^	3.50	1.022	0.687
2. The sooner I get the results, even when this is from a computer, the more I am at ease	3.37	1.014	− 0.657
3. Because of the use of artificial intelligence, fewer doctors and radiologists are required^2^	3.14	0.967	0.551
4. Evaluating scans with artificial intelligence will reduce healthcare waiting times^2^	2.44	0.736	0.404
5. In my opinion, humans make more errors than computers^2^	2.85	0.826	0.358
Factor 5, “being informed,” 4 items, Cronbach’s alpha 0.578, composite reliability 0.57			
Overall	3.31	0.703	
1. If it does not matter in costs, a computer should always make a full body scan instead of looking at specific body parts	3.88	1.052	0.621
2. If a computer would give the results, I would not feel emotional support	4.21	0.839	0.456
3. A computer should only look at body parts that were selected by my doctor	2.80	1.10	− 0.403
4. When a computer can predict that I will get a disease in the future, I want to know that no matter what	3.69	1.110	0.362

^1^Items measured on a 5-point scale (strongly disagree-strongly agree). For all factors higher scores indicate being more negative towards AI in radiology,

^2^Items marked are recoded to measure in the same direction.

^3^Bold printing of words as in original questionnaire

**Table 2:** Descriptive figures of 8 attitudinal items that were not included in one of the 5 factors

Attitudinal items	Mean^1^	Standard deviation
1. A computer should be able to find all unrequested incidental findings on a scan	4.32	0.567
2. Computers can deal with personal data more carefully than doctors^2^	3.28	0.797
3. It is impossible to address computers on their errors	4.18	0.867
4. It is clear to me who is responsible when a computer makes an error in evaluating a scan^2^	3.01	1.079
5. I find it no problem when a computer uses data from my scan and stores these for scientific research	3.86	1.025
6. Humans and artificial intelligence can complement each other	4.34	0.659
7. Human error is more harmful than error caused by computers	2.62	1.074
8. A computer is just a giant calculator	3.39	1.03

^1^Items measured on a 5-point scale (strongly disagree-strongly agree)

^2^Items marked are recoded to measure in the same direction within an original scale

**Table 3:** Correlations between factors

	Factor 1	Factor 2	Factor 3	Factor 4	Factor 5
Factor 1					
Pearson correlation	–	0.126	0.348**	0.224*	0.089
95% CI interval		(− 0.052, 0.296)	(0.182, 0.495)	(0.048, 0.386)	(− 0.089, 0.261)
Sample size		123	123	122	124
Factor 2					
Pearson correlation	0.126	–	0.161	− 0.096	0.029
95% CI interval	(− 0.052, 296)		(− 0.014, 0.327)	(− 0.266, 0.080)	(− 0.145, 0.202)
Sample size	123		126	125	127
Factor 3					
Pearson correlation	0.348**	0.161	–	0.192*	0.160
95% CI interval	(0.182, 0.495)	(− 0.014, 0.327)		(0.018, 0.355)	(− 0.014, 0.325)
Sample size	123	126		126	128
Factor 4					
Pearson correlation	0.224*	− 0.096	0.192*	–	0.140
95% CI interval	(0.048, 0.386)	(− 0.266, 0.080)	(0.018, 0.355)		(− 0.035, 0.307)
Sample size	122	125	126		127
Factor 5					
Pearson correlation	0.089	0.029	0.160	0.140	–
95% CI interval	(− 0.089, 0.261)	(− 0.145, 0.202)	(− 0.014, 0.325)	(− 0.035, 0.307)	
Sample size	124	127	128	127	

*p < 0.05 (2-tailed); ** *p* < 0.01 (2-tailed)

### Patients’ views on AI in radiology

The average score for factor 1 “distrust and accountability” was 3.28, which indicates that patients are moderately negative when it comes to their trust in AI in taking over diagnostic interpretation tasks of the radiologist, both with regard to accuracy, communication, and confidentiality. The average score for factor 2 “procedural knowledge” was 4.47, which indicates that patients are engaged in understanding how their imaging examinations are acquired, interpreted, and communicated. Patients also indicate to appreciate and prefer personal interaction over AI-based communication, with an average score of 4.38 for factor 3 “personal interaction.” In addition, patients were rather ambiguous as to whether AI will improve diagnostic workflow, given the average score of 2.89 for factor 4 “efficiency.” Within factor 5 “being informed,” scores on several items were not outspoken. For example, within this factor, patients tended to prefer AI systems to look at the entire body instead of specific body parts only (average score of 3.88) and to be informed by AI systems about future diseases they will experience when possible (average score of 3.69). On the other hand, patients indicated that they would feel a lack of emotional support when computers would provide them results (average score of 4.21).

### Associations of factors with other variables

Table 4 shows associations of factors with respondents’ characteristics. Factors 1 (“distrust and accountability”) and 3 (“personal interaction”) were significantly associated with inclination to change; the more respondents distrust AI in radiology (factor 1) or the more the respondents appreciate personal interaction, the lower their score on inclination to change (factor 1, *r* = − 0.39814, *p* < 0.01; factor 3, *r* = − 0.179, *p* < 0.5). Factor 1 was also significantly related to the education level of respondents; the level of trust steadily increased for each higher category in education level of respondents (*F*(4, 4) = 6.99, *p* < 0.01).

**Table 4:** Correlations and ANOVA of factors with demographic variables

	Factor 1	Factor 2	Factor 3	Factor 4	Factor 5
Age					
Pearson correlation	0.083	0.196*	0.050	− 0.200*	− 0.179
95% CI interval	(− 0.109, 0.269)	(0.022, 0.359)	(− 0.126, 0.223)	(− 0.363, − 0.025)	(− 0.343, − 0.005)
Sample size	122	126	126	125	127
Inclination to change					
Pearson correlation	− 0.398**	− 0.022	− 0.179*	0.008	0.117
95% CI interval	(− 0.537, − 0.238)	(− 0.195, 0.153)	(− 0.343, − 0.005)	(− 0.167, 0.183)	(− 0.058, 0.285)
Sample size	123	127	127	126	128
Education*gender					
*F*-value	0.758	1.915	2.156	0.325	1.481
df effect, df error	4, 108	4, 112	4, 112	4, 112	4, 113
Education					
*F*-value	6.99*	0.489	0.274	1.006	1.257
df effect, df error	4, 4	4, 4	4, 4	4, 4	4, 4
Gender					
*F*-value	5.12	0.649	1.300	3.528	2.338
df effect, df error	1, 15.72	1, 6.915	1, 6.599	1, 29.931	1, 8.028

*p < 0.05 (2-tailed); **p < 0.01 (2-tailed)

Factor 4 (“efficiency”) was weakly negatively associated with age (*r* = − 0.200, *p* < 0.05), which means that the older the respondents are, the less they think that AI increases efficiency, while factor 2 (“procedural knowledge”) was weakly positively associated with age (*r* = 0.196, *p* < 0.05). Gender was not significantly associated with any of the factors, nor did gender and education have significant interaction effects.

## Discussion

AI has advanced tremendously over the last years and is expected to cause a new digital revolution in the coming decades [21]. It is anticipated that radiology is one of the fields that will be transformed significantly. Many speculate about the potentially profound changes it will cause in the daily practice of a radiologist [22]. However, there is a lack of debate on how patients would perceive such a transformation. For example, would patients trust a computer algorithm? Would they prefer human interaction over technology? To the best of our knowledge, there are no studies on this topic in the literature.

In this study, we documented the development of a standardized questionnaire to measure patients’ attitudes towards AI in radiology. The questionnaire was developed on the basis of a previous qualitative study in a collaboration between radiologists and survey methodologists [3] and pretested for clarity and feasibility by means of cognitive interviews. Subsequently, 155 patients scheduled for CT, MRI, and/or conventional radiography on an outpatient basis filled out the questionnaire.

An exploratory factor analysis, which took several rounds in the selection of factors and items within each factor, revealed five factors: (1) “distrust and accountability of AI in radiology,” (2) “procedural knowledge of AI in radiology,” (3) “personal interaction with AI in radiology,” (4) “efficiency of AI in radiology,” and (5) “being informed of AI in radiology.” Two of these factors (“procedural knowledge” and “personal interaction”) almost exactly corresponded with the domains identified in the qualitative study [3]. For three factors (1, 2, and 3), the internal consistency was good (Cronbach’s alpha > 0.8); for one factor (4), it was acceptable (only just below 0.7); and for one factor (5), it was acceptable considering the lower number of items (*n* = 4) included (Cronbach’s alpha just below 0.6).

Some items of factor 5 loaded negatively, and although reverse coding easily solves this problem, it may also mean that items within this factor are multi-dimensional.

Factor 1 still included a large number of items. Since including many items will increase respondent burden, it may be worthwhile to reduce the number of items per scale, with preferably no more than 8 items per scale.

Thus, additional data collection with confirmatory factor analysis can be recommended to further refine the scale. Nevertheless, overall, the developed questionnaire provides a solid foundation to map patients’ views on AI in radiology.

Our findings with respect to associations between several demographic variables and trust and acceptance of AI are in line with earlier studies on acceptance of CHIT [22]. As Or and Kash [23] concluded in their review of 52 studies examining 94 factors that predict the acceptance of CHIT, successful implementation is only possible when patients accept the technology and, to this end, social factors such as subjective norm (opinions of doctors, family, and friends) need to be addressed.

Interestingly, the results of our survey show that patients are generally not overly optimistic about AI systems taking over diagnostic interpretations that are currently performed by radiologists. Patients indicated a general need to be well and completely informed on all aspects of the diagnostic process, both when it comes to how and which of their imaging data are acquired and processed. A strong need of patients to keep human interaction also emerged, particularly when communicating the results of their imaging examinations. These findings indicate that it is important to actively involve patients when developing AI systems for diagnostic, treatment planning, or prognostic purposes, and that patient information and education may be valuable when AI systems with proven value are to enter clinical practice. They also signify the patients’ need for the development of ethical and legal frameworks within which AI systems are allowed to operate. Furthermore, the clear need for human interaction and communication also indicates a potential role for radiologists in directly counseling patients about the results of their imaging examinations. Such a shift in practice may particularly be considered when AI takes over more and more tasks that are currently performed by radiologists. Importantly, the findings of our survey only provide a current understanding on patients’ views on AI in general radiology.

The developed questionnaire can be used in future time points and in more specific patient groups that undergo specific types of imaging, which will provide valuable information on how to adapt radiological AI systems and their use to the needs of patients.

Limitations of our study include the fact that validation was done by means of cognitive interviews and exploratory factor analysis, which may be viewed as subjective. Validation with other criteria, such as comparison with existing scales, was not possible due to unavailability of such scales. Furthermore, our questionnaire was tested in patients on an outpatient basis, which may not be representative of the entire population of radiology patients.

In addition, although we explored the acceptability of purely AI-generated reports with patients, the acceptability of radiologist-written, AI-enhanced reports, which may well be the norm in the future, was not addressed.

It should also be mentioned that we did not systematically record the number and reasons of patients who were not able or refused to participate. Nevertheless, in the far majority of patients who did not participate, this was due to a lack of time.

In conclusion, our study yielded a viable questionnaire to measure acceptance among patients of the implementation of AI in radiology. Additional data collection may provide further refinement of the scale.

## Abbreviations and acronyms

AI: Artificial intelligence
CHIT: Consumer health information technology
EFA: Exploratory factor analysis
TAM: Technology acceptance model
